# Hypothesis: A modern human range expansion ~300,000 years ago explains Neandertal origins

**DOI:** 10.64898/2026.03.11.711219

**Published:** 2026-03-13

**Authors:** David Reich

**Affiliations:** 1 Department of Human Evolutionary Biology, Harvard University, Cambridge, MA, USA; 2 Department of Genetics, Harvard Medical School, Boston, MA, USA; 3 Howard Hughes Medical Institute, Harvard Medical School, Boston, MA, USA; 4 Broad Institute of MIT and Harvard, Cambridge, MA, USA

## Abstract

This paper demonstrates the feasibility of the hypothesis that Neandertals formed when a population using recently developed Levallois stone tool technology expanded between 400–250 thousand years ago (ka). In Europe, their range expansion into an area with Sima de los Huesos-like people led to massive introgression of local archaic genes producing a population with around 95% archaic ancestry (Neandertals); if this range expansion was sex-biased it would provide a simple explanation for why Neandertals retain modern human lineage Y chromosomes or mitochondrial DNA. In Africa, interbreeding with local archaic humans led to more modest archaic admixture and the deep substructure detected in all modern humans today. This proposal explains four previously perplexing similarities of modern humans and Neandertals—sharing of mitochondrial DNA, Y chromosomes, Levallois tools, and 300–200 ka date of formation by mixture—even while Neandertals and Denisovans cluster genome-wide.

## Introduction

**Figure 1:**
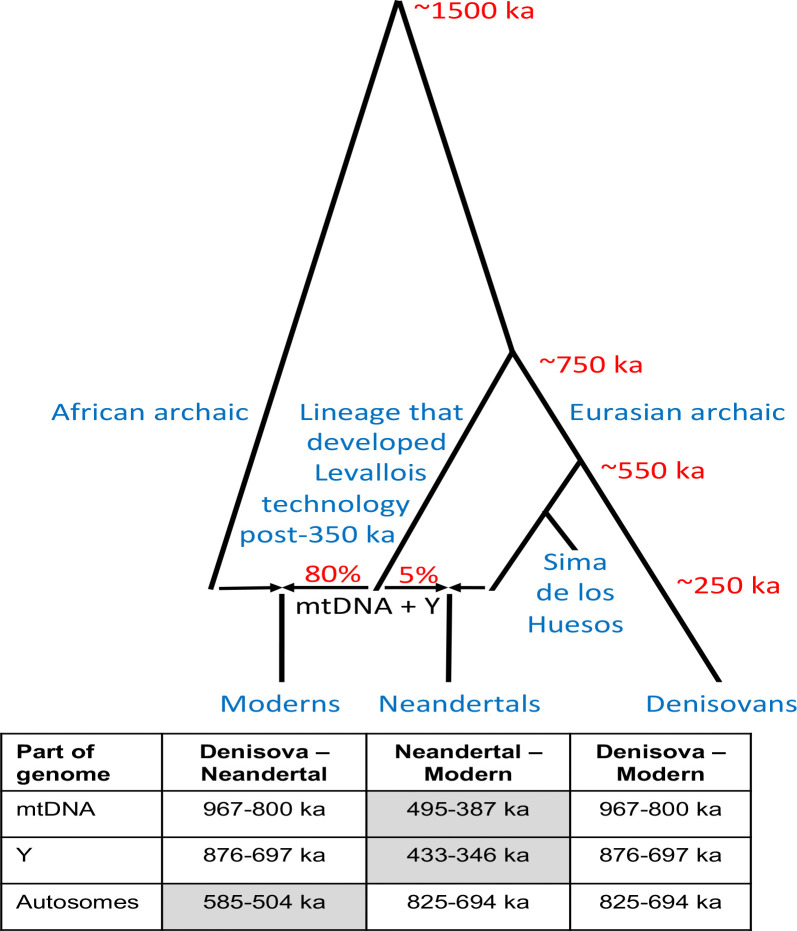
Archaic-modern relationships. Neandertals and Denisovans are sister groups; modern humans more distantly related; there is deep substructure in Africa; gene flow ~300–200 ka into Eurasian archaics to produce Neandertals; and simultaneously to African archaics to produce moderns. I do not show the superarchaic lineage contributing a few percent to Denisovans. At bottom are 95% confidence intervals for genetic divergences and population splits^1^; gray highlighting indicates the closer pair.

Neandertals and Denisovans derive the great majority of their nuclear genomes from a shared ancestral population that lived 585–504 thousand years ago (ka), which in turn diverged from a common ancestral population with modern humans 825–694 ka (Figure 1; all 95% confidence intervals from ref. 1). This reconstruction is consistent with the estimated split times of Denisovans and modern humans on the Y chromosome (876–697 ka) and mitochondrial DNA (mtDNA, 967–800 ka). But multiple analyses have revealed gene flow from the lineage leading to modern human into the ancestors of Neandertals hundreds of thousands of years later. Evidence comes from the estimated split time of Neandertals and modern humans in mtDNA (495–387 ka)^2^ and on the Y chromosome (433–346 ka)^3^, both of whose 95% confidence intervals^1^ are less than the inferred Neandertal-modern human split from autosomal data: impossible if there was not a later gene flow event. Analysis of haplotype patterns in the nuclear genome also estimated that Neandertals derived about 5% ancestry from admixture with modern humans at this time^4,5,6^, giving an estimate of 300–200 ka^5^ consistent with the mtDNA and Y evidence. Finally, DNA from the Sima de los Huesos individual from Spain estimated to have lived 430–300 ka^7^ revealed mtDNA related to Denisovans, but a nuclear genome on the lineage leading to Neandertals^8,9^. This is as expected if Neandertal ancestors were produced by introgression 300–200 ka of the modern human lineage into archaic populations like that of Sima de los Huesos, displacing only a fraction of its nuclear genome but ultimately all its mtDNA.

But this model is implausible in its simplest version: one that proposes no natural selection, and a one-time interbreeding event bringing genes from modern humans into Neandertals (Figure 1). First, in the absence of natural selection, the probability that both the modern human mtDNA and Y chromosome swept to high frequency by chance for a five percent introgression is only 0.25% = 5% × 5%. Second, if one resolves this conundrum by proposing that there was selection at both the Y chromosome and mtDNA for alleles derived from the modern human rather than the archaic lineage^3^, this is opposite to the typical pattern in the rest of the genome, where there is deficiency of modern human DNA in biologically significant regions^4,5,6^, consistent with findings that hybridizations tend to discriminate against functionally important alleles from the minority admixing population (due to co-evolved genetic variants at different loci which only work effectively when both are from the same population: Dobzhansky-Muller incompatibilities)^10^. Third, the Y chromosome and mtDNA, one transmitted by father the other by mother, could not have more different inheritance patterns, making it challenging to explain the patterns at both loci by sex-biased demography or selective processes. Thus, while there are numerous ecological examples where mitochondrial DNA patterns mismatch those of the rest of the genome potentially reflecting natural selection or sex-biased admixture^11,12,13^, or a rare allele (around 5% in this case) driven to high frequencies due to genetic drift, it seems unlikely for both mtDNA and Y.

I propose an explanation inspired by the literature on genetic consequences of range expansions in diverse species. When a new population moves into territory occupied by a previously established one and outcompetes it, massive introgression of local genes often occurs, so that by the time the new population finishes spreading, its genetic composition can be largely local^14,15^. I hypothesize that such a range expansion occurred 400–250 ka: more recent than the 95% confidence intervals for the Y chromosome and mtDNA split times which places an upper bound on when modern humans and Neandertals last shared ancestors, and older than the estimated admixture dates of modern and archaic lineages both in Eurasia (around 300–200 ka) and in Africa (around 300 ka^5,16^). This is within the time period when a new tool production technique (Levallois technology) based on prepared stone cores spread across a wide area of Africa where it was used by modern humans (known there as the Middle Stone Age and starting by at least 400 ka^17^), and also in the Middle East and Europe where it was used by Neandertals (known there as the Middle Paleolithic, starting there by at least 480–300 ka^18^). There is debate about whether Levallois technology, which never spread to southern or eastern Asia, was invented once or multiple times^19^, but it is notable that the period around 500–350 ka corresponds not just to Levallois, but also to other cultural advances that increase the likelihood of a spread of people around this time, including improved hafting technology, and a dramatic expansion in the use of fire^20^. People who mastered these technologies would plausibly have been able to exploit local resources more effectively, maintaining a higher density in most areas where they lived than the previous archaic lineage groups. As populations with these traits expanded to Europe, range expansion theory predicts they would have experienced massive introgression of local archaic genes from archaic pre-Neandertal populations like those at Sima de los Huesos sharing genetic drift with later Neandertals. If the new expanding population passed its cultural package to the next generation matrilineally^21^ (with local archaic men but few women incorporated into the expanding population), or patrilineally, this would provide a simple explanation for why one of the two uniparentally inherited alleles persisted in later Neandertals, eliminating the need to hypothesize natural selection for both modern human lineage mtDNA and Y chromosomes. This leaves the conundrum of why the other uniparentally transmitted allele in Neandertals is not from the archaic lineage, since a constant incorporation of local archaic people of one sex would have added either archaic Y chromosome or mtDNA. However, this is far more plausible than at two loci, and there are multiple possible explanations as I discuss in what follows.

## Simulation setup

**Figure 2:**
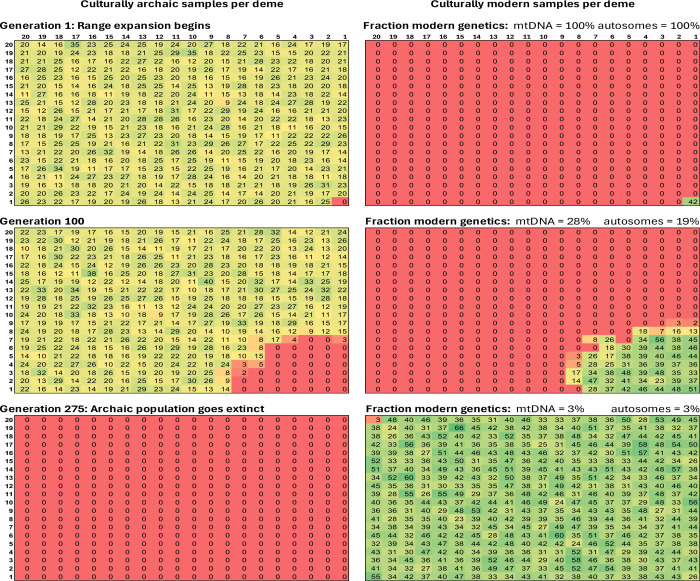
A typical simulation showing the wave of advance. Baseline setting is *K*_*A*_=20, *K*_*M*_=40, *m*=0.05, γ=0.15, *r*=0.5, 20×20 demes, *s*=0, and no Dobzhansky-Muller incompatibilities. Counts per deme are shown after Poisson sampling of offspring at the end of the generation.

I wrote a computer simulation inspired by previous work that considered how range expansions impact patterns of genetic variation^14,15^. The simulation approximates Europe as a grid of 20×20 demes, each seeded with *N*_*A,i,j,1*_ *= K*_*A*_ archaic individuals (*N*_*A,i,j,t*_ is the number of archaic individuals in deme (*i, j*) at generation *t*, and *K*_*A*_ is the archaic carrying capacity per deme) (Figure 2). The exception is the lower right deme *(1, 1)*, which is set as having no archaic humans, and instead is seeded with *N*_*M,1,1,1*_ *= K*_*M*_ modern lineage individuals (*N*_*M,i,j,t*_ is the number of modern lineage individuals in deme (*i, j*) at generation *t*, and *K*_*M*_ the modern lineage carrying capacity set to be larger than *K*_*A*_). Individuals are determined as male with 50% probability.

In each generation, the simulation iterates through four steps. Below I specify a patrilineal range expansion, but mathematical symmetry means the results are the same for a matrilineal expansion.

- Migration: Each individual has a chance to move one deme in an adjacent direction, if it is not off the edge of the grid (probability *m* in each direction).
- Interbreeding (females leaving their natal population): Each archaic female changes their identity from archaic to modern with probability *γN*_*M,i,j,t*_/(*γN*_*M,i,j,t*_+*N*_*A,i,j,t*_), and vice versa with probability *γN*_*A,i,j,t*_/(*γN*_*A,i,j,t*_+*N*_*M,i,j,t*_). *γ* captures assortative mating: the reduced probability of a female mating with a male of the other than with one of the same population. The full expression adjusts for the relative number of males: if there are more males from one group, this proportionally increases the probability that a female in the deme will mate with such a male.
- Determination of the number of individuals in generation *t+1*: The expected number of archaic and modern humans in the next generation is E[*N*_*A,i,j,t+1*_]=*N*_*A,i,j,t*_(1+*r*(*K*_*A*_-*N*_*A,i,j,t*_*-N*_*M,i,j,t*_)/(*N*_*A,i,j,t*_+*N*_*M,i,j,t*_) and E[*N*_*M,i,j,t+1*_]=*N*_*M,i,j,t*_(1+*r*(*K*_*M*_-*N*_*A,i,j,t*_*-N*_*M,i,j,t*_)/(*N*_*A,i,j,t*_+*N*_*M,i,j,t*_). This captures the expectation that there will be logistic growth (or contraction) at intrinsic rate *r* to match the deme’s carrying capacity. These expressions imply that modern individuals will eventually outcompete archaic ones within a deme: if the number of individuals *N*_*A,i,j,t*_+*N*_*M,i,j,t*_ is greater than *K*_*A*_ but less than *K*_*M*_, the number of archaic individuals in the next generation is expected to shrink and the number of modern individuals grow. Both a male and a female from a population are needed to reproduce, so I set the expectation for the number of individuals in generation *t+1* to be zero if there are either zero males or females from that population in generation *t*. I determine the number of individuals in the next generation by Poisson sampling, and I assign them to be male with 50% probability. If the lower right deme (where expansion starts) is sampled by chance to have either zero modern lineage males or females in any generation, these are repopulated into the deme.
- Mating: For each individual in generation *t+1*, I randomly sample parents from males and females of the previous generation *t*. I simulate ten unlinked loci: mtDNA (from the mother), Y chromosome (from the father), and autosomal loci 1–8, where the allele transmitted to the offspring is randomly sampled from the two the parents carries. The transmitted alleles are added to produce a diploid genotype. I simulation selection against archaic mtDNA in one of two ways:
  1. *Basic*: If the simulated offspring has archaic mtDNA, they are removed with probability *s*, and new parents randomly sampled. This is repeated until parents are successfully chosen.
  2. *Dobzhansky-Muller incompatibility*: If the simulated offspring has archaic mtDNA, and the first autosomal locus is homozygous modern, they are removed with probability *s*, and new parents are randomly sampled. This is repeated until parents are successfully chosen.

This simulation is an oversimplification. For example, Europe is not a square grid with every part of the continent equally habitable. I chose a simplified simulation because my goal is an existence proof: to demonstrate the plausibility of massive introgression of local genes on the autosomes with a qualitatively different behavior on both the Y chromosome and mtDNA. I did not want to make it seem that these results were dependent on knowing accurately the details of the essentially unknowable demography of humans who lived in several hundred thousand years ago.

## Simulation results

Figure 2 shows how the counts of archaic and modern individuals per deme (with population affiliation determined based on who a person’s father is) evolves over time. The specific simulation shows baseline settings of *K*_*A*_=20, *K*_*M*_=40, *m*=0.05, γ=0.15, r=0.5, dimension of the simulated landscape 20 (20×20 demes), *s*=0, and no Dobzhansky-Muller incompatibilities. I chose the carrying capacities so that the simulated landscape has a census size that is not unreasonable based on reconstructions of human population sizes in the past: 8000 for archaic humans, and 16000 for modern humans (effective sizes of thousands or tens of thousands are typically inferred^1^). The results replicate the qualitative behavior of previously reported simulations of range expansions^14^, showing a wave-of-advance. Behind the wavefront there are only modern humans, beyond it only archaic humans, and at the wavefront, there are people from both populations (Figure 2). The modern humans at the wavefront are the fastest growing subgroup in the simulated landscape. It is these modern lineage individuals, initially outnumbered by archaic humans in their deme, who are exposed to substantial gene flow from archaic humans. Their rapid population growth amplifies the archaic DNA they take on through admixture, so even after the first hundred generations more than half their genetic ancestry is typically archaic (Figure 2). Compounding this process deme-by-deme as the wavefront sweeps across the landscape, means that by the end of the simulation, there is typically only a small fraction of modern human lineage-derived genes left (Figure 2), despite the population retaining modern human material culture (for example Levallois technique).

**Figure 3:**
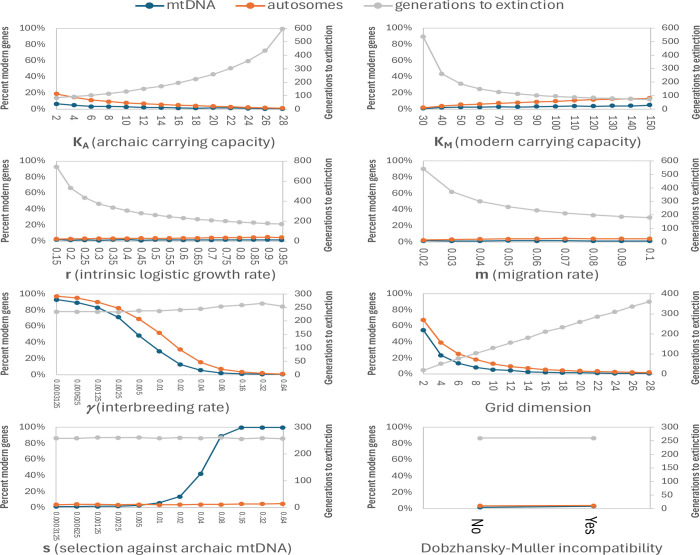
Simulation results. Baseline setting is *K*_*A*_=20, *K*_*M*_=40, *m*=0.05, γ=0.15, r=0.5, dimension of the simulated landscape 20, *s*=0, and no Dobzhansky-Muller incompatibilities. Results show an average of 100 simulations for each parameter combination.

Since the true parameters of the demographic history are unknown, I also varied each simulation parameter from the baseline in turn, and tracked four quantities: (a) the number of generations until archaic population extinction (defined as the generation when the last archaic Y patrilines disappear), (b) the proportion of modern ancestry on the autosomes (summarized as the average of autosomal loci 2–8 (excluding locus 1 as in some simulations it participates in Dobzhansky-Muller incompatibilities), (c) the proportion of modern ancestry at mtDNA and (d) at the Y. I verified that the Y chromosome is always 100% modern human after archaic extinction, as expected from a patrilineal setup. The simulations confirm that for a wide range of parameters, there is massive introgression of archaic genes^14,15^ (Figure 3).

One of the most consistent features of the simulations is that when there is massive introgression of local archaic genes affecting the autosomes, mtDNA is almost always archaic as well (Figure 3). This aligns with the intuition that the observed pattern of a small proportion of modern human lineage ancestry on the autosomes, and 100% modern human lineage at mtDNA, is surprising. However, the simulations can match the observed pattern of mitochondrial - nuclear discordance in ancestry if there has been selection for modern human lineage mtDNA, as long as its fitness advantage relative to archaic mtDNA is at least than around 5% (Figure 3). I also tried simulating Dobzhansky-Muller incompatibilities between mtDNA and autosomal locus 1, but I found no combination of parameters for which I was able to decouple the fixation probability between the nuclear genome and mtDNA: in all my simulations, the effect of such a simulated incompatibility was to rapidly drive down archaic ancestry at autosomal locus 1 quickly to zero. Dobzhansky-Muller incompatibilities are well-known in hybridization events and often involve mtDNA; they are an appealing explanation for a selective process that drove the archaic mtDNA to extinction so it is important to continue to search for scenarios where they could be involved.

I explored the possibility that the rise to 100% frequency of the modern human lineage mtDNA (and/or Y chromosome) in Neandertals could have arisen due to the buildup of slightly deleterious mutations which is expected in theory at loci not broken up by recombination^22^. This process is expected to proceed more quickly on lineages with smaller population sizes, and indeed the archaic lineage of Neandertals is estimated to have been ~2- to 7-fold smaller the modern human lineage on average since they diverged^1,16^. However, my simulations of this process found that it is likely to have been too slow to produce a >5% fitness difference of mtDNA sequences on the two lineages (Extended Data Figure 1). This is consistent with empirical work showing no substantial differences in fitness across mtDNA sequences of divergent primates, birds and fruit flies^23^.

## A unified explanation for four previously perplexing observations

This model of a unilineal modern human range expansion is conceptually as simple as the classic model of one-time introgression of modern into archaic humans. But it provides a match to multiple observations not explained by the classic model.

First, unlike the classic model, the new model is not strained to explain the evidence of ~5% of the nuclear genome having an origin in one ancestral population, combined with 100% of Y and mtDNA from the other, a genetic pattern that I am not aware of being observed in diverse natural natural contexts. Second, range expansion of modern humans through Europe is expected to lead to massive introgression of local genes^14,15^, consistent with the data. Third, the model has archaeological explanatory power, resolving the conundrum of why Neandertals and modern humans shared Levallois technology^19,20^. This suggests that in some respects, an appropriate way to view Neandertals may not be as archaic group, but instead as a group that retained important modern human features even in the face of displacement of most modern human lineage genes.

This model also sheds light on an independent set of findings regarding deep population structure in Africa. Multiple studies have inferred that the ancestors of all modern humans including sub-Saharan Africans were deeply substructured, due to the coming together a few hundred thousand years ago of lineages that began diverging a million or more years ago^16,24,25^. For example, ref. 16 models modern human ancestry as largely derived from a mixture of about 80% from a lineage that was most closely related to Neandertals and Denisovans, and 20% from a lineage that diverged from it around 1.5 million years ago, with the two coming together around 300 ka. The remixture is estimated to date to around the same time as the genetically inferred interbreeding of modern humans and archaic humans in the ancestors of Neandertals 300–200 ka. This raises the possibility that these mixtures in Europe and Africa had related causes: a range expansion of a successful population interbreeding with local archaic groups. In Europe, there was massive introgression of local genes because barriers to producing viable offspring were few. In Africa, the mixture was with a more divergent archaic lineage, result in a lower γ (cross-group interbreeding rate), and less introgression. Table 1 shows four features of real data that are explained by this proposed model.

## Open questions

Was the 400–250 ka range expansion patrilineal or matrilineal? Patrilineality with incorporation of archaic females into expanding modern human groups is plausible on behavioral grounds, as female dispersal and males remaining in their natal groups is predominant in great apes as well as in diverse humans today^26^. Such a scenario would explain why the modern human lineage Y chromosome was retained even in the face of streams of incoming archaic females who were sufficient to displace most modern human lineage ancestry on the autosomes. An explanation would then still be required for retention of modern human lineage mtDNA. However, my simulations in Figure 3 showed that if Denisovan/Sima mtDNA had at least around 5% reduced fitness relative to Neandertal/modern human mtDNA, retention of the modern human lineage mtDNA would be expected^3^ (Figure 3). Experimental evidence for differential fitness of the two mtDNA types could, in principle, be obtained through gene editing experiments where human cell lines were created with mtDNA of either the Denisovan or the Neandertal/modern human type, then tested for measures of mitochondrial function. A challenge to such experimental tests is that if the fitness effects varied in different cell lines and environments, it would be challenging to make a confident statement about how this would manifest at a whole organism level in ancient humans. If the phenotypic events were far stronger—as expected for Dobzhansky-Muller incompatibilities between mitochondrial DNA and nuclear interacting genes which frequently been able to show physiological effects of mitochondrial - nuclear ancestry mismatch including in tests of isolated mitochondria^27,28,29,30^—a strong phenotype could be easy to detect.

A matrilineal range expansion with incorporation of archaic males into modern human groups is also plausible^31^. Males have more variation in reproductive success than females, and if females prefer mates whose fathers were from the modern population, this would rapidly remove introgressing archaic Y chromosomes without there having to be reduced biological fitness associated with archaic Y chromosomes. In fact, a model of a matrilineal human range expansion has some empirical support based on estimates of more Neandertal introgressed segments on the X chromosome than the autosomal average^5,21^: a recent study that fit the measured excess on the X chromosome to numerical simulations found that such a pattern could only be explained by modern humans females mating with a stream of archaic males to the point that most of the nuclear genome was displaced and a smaller proportion of the X chromosome^21^, exactly the matrilineal case. A caution is that the evidence of an X-chromosome excess of modern human admixture into Neandertals is not compelling: four non-sex chromosomes have even higher modern human ancestry estimates than on the X chromosome^5^; methods for estimating modern human introgression into Neandertals suffer from high false-negative rates which are likely to be different on the autosomes and chromosome X due to their fundamentally different patterns of variation; and natural selection against variants on chromosome X is likely to follow a different pattern than that on the autosomes. All of these patterns make it challenging to compare reliably the estimates of modern human introgression in these two parts of the genome^5^, so at present I view both patrilineal and matrilineal scenarios as compatible with the data.

A common way to search for distinctively modern human genetic innovations has been to search for alleles common in all living people, but absent in the Neandertal genome^32^. But if the proposed model is right and a useful way to view Neandertals is as early modern humans who were massively introgressed with local genes, then multiple loci in the genome that are biologically important for modern humans might be expected to be shared between Neandertals and living people. It is therefore of interest to understand the biological impact of variants where all Neandertals and modern humans share common ancestors on the order of a few hundred thousand years ago, while Denisovans are much more distantly related. Previous work has shown that there are only on the order of a million bases of the alignable genome that are candidates for these loci with all modern human genomes sharing a common ancestor within the last half million years^33.^ The subset where Neandertals share the recent coalescence, but Denisovans do not, is smaller still. Screening for places where all modern humans and Neandertals share an ancestor ~700–300 ka thus has the potential to pinpoint alleles important in distinctively modern biology.

## Extended Data

**Extended Data Figure 1 –:**
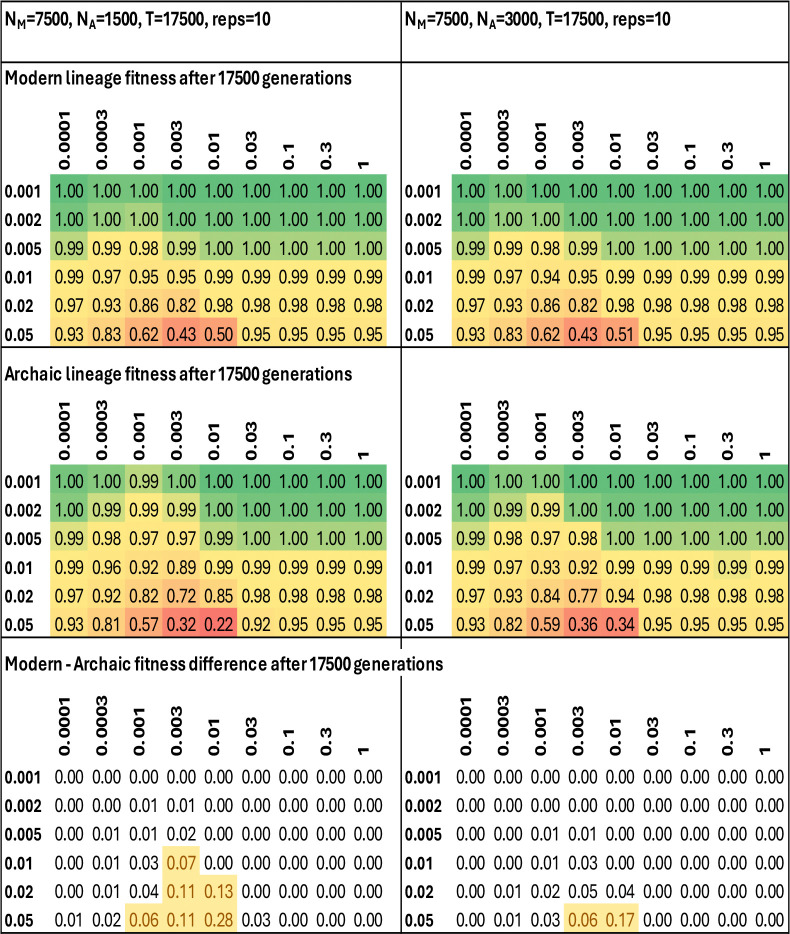
Simulations to test if differences in archaic and modern populations sizes could explain reduced mtDNA fitness of archaic lineages. I assumed: (a) The archaic and modern lineages ancestral to Neandertals split ~750 ka and remixed ~250 ka, corresponding to T=17500 generations of separation assuming 28.6 years per generation. (b) The modern lineage population size was constant at N_M_=7500 (female population size is what matters for mtDNA; I used half the ~15000 diploid population size estimated in Supplementary Figure 10 of ref. 16, similar to the 11000–16000 in Figure 4 of ref. 1). (c) The archaic population size N_A_ was also constant and 1/2 to 1/7 of the modern lineage (factor based on Figure 4 of ref. 1). I initialized all samples in the modern (N_M_=7500) or archaic (N_A_=1500 or 3000) population to fitness *f*_*i*_=1 (*i* is the sample index). I added a mutation to each individual with probability *μ* (*μ* was fixed for each simulation), and assigned all such mutations to be selected against with coefficient *s* (*s* was fixed to be the same for all mutations for each simulation), updating fitness to *f*_*i*_=(1-*s*)*f*_*i*_. I obtained the next generation by sampling parents from the previous one, resampling with probability 1-*f*_*i*_ (this captures the expectation that parents with lower fitness have a reduced probability of reproducing). I show the average fitness across all mtDNA sequences in the final generation, further averaged over 10 replicates, for: modern human lineage fitness, archaic lineage fitness, and difference in fitness. For the difference in fitness, I highlight in yellow values above 5%, the approximate point at which the simulations show the mtDNA is expected to drive to fixation (Figure 3). mtDNA has an estimated coding sequence mutation rate of 2.87×10^−6^ mutations per base pair per generation over about 15400 bases of coding sequence^34^, translating to an estimated rate of 0.044 coding mutations per mtDNA genome per generation, close to the maximum μ I simulated. However, it is only between selection coefficients of 0.001–0.01 that there is expected to be a substantial differential buildup of deleterious mutations, and only a small fraction of newly arising mutations are likely to have *s* in this range. The rate of newly arising mutation in this range needs to be at least 0.01 per mtDNA per generation to produce an expected fitness reduction greater than 5%, which is high enough that it is perhaps not surprising that there is no empirical evidence for this process leading to substantial differences in mtDNA fitness across diverse species pairs^23^.

## Supplementary Material

Supplement 1

## Figures and Tables

**Table 1:** Four features of real data explained by the proposed model

Shared Neandertal and modern human trait not in Denisovans	Predicted by model of unilineal range expansion	Predicted by one-time mixture model

Y	Yes	No
mtDNA	Yes	No
300–200 ka mixture	Yes	No
Levallois tools	Yes	No

## Data Availability

No new data are reported.

## Code Availability Statement

The code used to generate the results in Figures 2 and 3 as well as Extended Data Figure 1 is available as supplementary files.

## References

[1] Peyrégne S (2025) A high-coverage genome from a 200,000-year-old Denisovan. bioRxiv 2025.10.20.683404.

[2] Posth C (2017) Deeply divergent archaic mitochondrial genome provides lower time boundary for African gene flow into Neandertals. Nat Commun. 8, 16046. 28675384 10.1038/ncomms16046 PMC5500885

[3] Petr M (2020) The evolutionary history of Neandertal and Denisovan 4mosomes. Science 369, 1653–6. 32973032 10.1126/science.abb6460

[4] Kuhlwilm M (2016) Ancient gene flow from early modern humans into Eastern Neandertals. Nature 530, 429–433 (2016). 26886800 10.1038/nature16544 PMC4933530

[5] Hubisz MJ, Williams AL, Siepel A (2020) Mapping gene flow between ancient hominins through demography-aware inference of the ancestral recombination graph. PLoS Genetics 16, e1008895. 32760067 10.1371/journal.pgen.1008895 PMC7410169

[6] Harris DN (2023) Diverse African genomes reveal selection on ancient modern human introgressions in Neandertals. Current Biology 33, 4905–16. 37837965 10.1016/j.cub.2023.09.066 PMC10841429

[7] Arsuaga JL (2024) How the Sima de los Huesos was won. Anat Rec 307, 2225–45. 10.1002/ar.25509 38813936

[8] Meyer M (2014) A mitochondrial genome sequence of a hominin from Sima de los Huesos. Nature 505, 403–6. 24305051 10.1038/nature12788

[9] Meyer M (2016) Nuclear DNA sequences from the Middle Pleistocene Sima de los Huesos hominins. Nature 531, 504–7. 26976447 10.1038/nature17405

[10] Langdon QK (2024) Swordtail fish hybrids reveal that genome evolution is surprisingly predictable after initial hybridization. PLoS Biol 22, e3002742. 39186811 10.1371/journal.pbio.3002742 PMC11379403

[11] Alves PC, Melo-Ferreira J, Freitas H, Boursot P. The ubiquitous mountain hare mitochondria: multiple introgressive hybridization in hares, genus Lepus. Philosophical Transactions of the Royal Society of London. Series B, Biological Sciences 363, 2831–9. 10.1098/rstb.2008.0053 PMC2606744 18508749

[12] Boratyński Z, Melo-Ferreira J Alves P (2014) Molecular and ecological signs of mitochondrial adaptation: consequences for introgression? Heredity 113, 277–86. 24690754 10.1038/hdy.2014.28 PMC4181067

[13] Roca AL, Georgiadis N, O’Brien SJ (2025) Cytonuclear genomic dissociation in African elephant species. Nat Genet. 37, 96–100. 10.1038/ng1485 15592471

[14] Currat M and Excoffier L (2004) Modern humans did not admix with Neandertals during their range expansion into Europe. PLoS Biol. 2, e421. 15562317 10.1371/journal.pbio.0020421 PMC532389

[15] Currat M, Ruedi M, Petit RJ, Excoffier L (2008) The hidden side of invasions: Massive introgression by local genes. Evolution 62, 1908–20. 18452573 10.1111/j.1558-5646.2008.00413.x

[16] Cousins T, Scally A, Durbin R (2025) A structured coalescent model reveals deep ancestral structure shared by all modern humans. Nat Genet 57, 856–864. 40102687 10.1038/s41588-025-02117-1 PMC11985351

[17] Shipton C (2022) Predetermined Refinement: the Earliest Levallois of the Kapthurin Formation. Journal of Paleolithic Archaeology 5, 4.

[18] Moncel MH (2020) Early Levallois core technology between Marine Isotope Stage 12 and 9 in Western Europe. J Hum Evol 139, 102735. 32078934 10.1016/j.jhevol.2019.102735

[19] Adler DS (2014) Early Levallois technology and the Lower to Middle Paleolithic transition in the Southern Caucasus. Science 345, 1609–13. 25258079 10.1126/science.1256484

[20] Kuhn SL (2020). The Evolution of Paleolithic Technologies (1st edition). Routledge.

[21] Platt A, Harris DN, Tishkoff SA (2026) Interbreeding betweenNeanderthals and modern humans was strongly sex biased. Science, 391, 922–5. 41747031 10.1126/science.aea6774 PMC13345703

[22] Muller HJ (1964). The relation of recombination to mutational advance. Mutation Research/Fundamental and Molecular Mechanisms of Mutagenesis, 1, 2–9. 10.1016/0027-5107(64)90047-8 14195748

[23] Mo YK, Mishra SR, Peña-Garcia Y, Hahn MW (2025) No molecular evidence for Muller’s ratchet in mitochondrial genomes. bioRxiv 2025.12.16.694700.

[24] Durvasula A, Sankararaman S (2020) Recovering signals of ghost archaic introgression in African populations. Sci. Adv. 6, eaax5097. 32095519 10.1126/sciadv.aax5097 PMC7015685

[25] Ragsdale AP (2023) A weakly structured stem for human origins in Africa. Nature 617, 755–763. 37198480 10.1038/s41586-023-06055-y PMC10208968

[26] Koenig A, Borries C. (2012), Hominoid dispersal patterns and human evolution. Evol. Anthropol., 21, 108–112. 22718478 10.1002/evan.21300

[27] Burton RS, Barreto FS (2012) A disproportionate role for mtDNA in Dobzhansky-Muller incompatibilities? Mol Ecol. 21, 4942–57. 22994153 10.1111/mec.12006

[28] MA H (2016) Incompatibility between nuclear and mitochondrial gnomes contributes to an interspecies reproductive barrier. Cell Metabolism, 24, 283–94. 27425585 10.1016/j.cmet.2016.06.012 PMC4981548

[29] Lima T, Burton RS, Willett CS (2019) Genomic scans reveal multiple mito-nuclear incompatibilities in population crosses of the copepod *Tigriopus californicus*. Evolution, 73, 609–20. 30675905 10.1111/evo.13690

[30] Moran BM (2024) A lethal mitonuclear incompatibility in complex I of natural hybrids. Nature 626, 119–27. 38200310 10.1038/s41586-023-06895-8 PMC10830419

[31] Jones D (2011) The matrilocal tribe: an organization of demic expansion. Hum Nat. 22, 177–200. 22388807 10.1007/s12110-011-9108-6

[32] Green RE (2010) A Draft Sequence of the Neandertal Genome. Science 328, 710–22. 20448178 10.1126/science.1188021 PMC5100745

[33] Mallick SM (2016) The Simons Genome Diversity Project: 300 genomes from 142 diverse populations. Nature 538, 201–6. 27654912 10.1038/nature18964 PMC5161557

[34] Árnadóttir ER (2024) The rate and nature of mitochondrial DNA mutations in human pedigrees. Cell, 187, 3904–18. 38851187 10.1016/j.cell.2024.05.022

